# Supplementary material for: Phase I dose escalation of the Exportin 1 inhibitor, Selinexor, in combination with chemoradiation in patients with newly diagnosed glioblastoma
Source: medRxiv. 2026 Jul 7:2026.06.25.26356263. Preprint. [Version 1] doi: 10.64898/2026.06.25.26356263 (PMC13370515; doi:10.64898/2026.06.25.26356263)
Supplement: Supplementary file 1 [file NIHPP2026.06.25.26356263V1-supplement-1.pdf]

Supplemental Table 1

EORTC Volumes	
GTV	Cavity + enhancing tumor
CTV	GTV + 2cm
PTV	CTV + 3-5mm
RTOG Boost Volumes	
GTV	Cavity + enhancing tumor
CTV	GTV + 2cm
PTV	CTV + 3-5mm
Organ at Risk	Objective
Brainstem	Dmax < 60Gy
Chiasm	Dmax < 55Gy
Cochlea	mean < 45 Gy
Eyes	Macula < 45Gy
Optic nerves	Dmax < 55 Gy
RT Volumes to 60Gy	
Median GTV, cc (range)	24.2 (7.5-40.7)
Median CTV, cc (range)	162.7 (75.7-300.2)
Median PTV, cc (range)	222.3 (116.1-388.2)
